# PW01-034 – Clinical-genetic investigation of FMF in Armenia

**DOI:** 10.1186/1546-0096-11-S1-A87

**Published:** 2013-11-08

**Authors:** H Hayrapetyan, G Amaryan, A Yeghiazaryan, T Sarkisian

**Affiliations:** 1 Center of Medical Genetics and Primary Health Care, Yerevan, Armenia; 2 Arabkir Joint Medical Centre - Institute of Child and Adolescent Health, Yerevan, Armenia

## Introduction

Familial Mediterranean Fever (FMF) is the most common hereditary disorder among Armenians. The establishment of clinical and genetic testing of FMF in the Center of Medical Genetics and Primary Health Care (CMG) was mostly forced by the high social and public health problems concerning a huge cohort of patients.

## Objectives

Prevalence of FMF is 14-100/per 10000 in different regions of Armenia. Frequency of carriers of MEFV mutations is 1:3 in Armenians. And, the increase of the incidence of FMF and related disorders is caused by genetic drift and geographical isolation.

## Methods

Molecular genetic detection of 12 MEFV mutations accounting for 98,71% of patients compared to healthy individuals revealed the most frequent genotypes and genotype-phenotype correlations.

## Results

Heterozygote carriers associated with abortive and mild FMF features is 18,72%, and 1.29% of patients with clinical features of FMF are without mutations. In some FMF patients “mild” MEFV mutations are associated with inflammatory attacks (P369S: 0.49%; E148Q: 5.09%; A744S: 0.74%). Genotypes E148Q/A744S and E148Q/P369S are found rarely.

We have revealed the complex FMF cases with following concurrent morbidity: epilepsy (M694V/M694V; V726A/M680I); Sjogren syndrome (M694V/M694V); bronchial asthma (M694V/V726A, V726A/M680I, M680I); b-thalassemia (M694V/M694V); hyperthyroidism (M694V/M680I); Tourette syndrome (M694V/M694V); Ulcerative colitis (M694V/M694V); renal amyloidosis and multiple sclerosis (M680I/M680I); ankylosing spondilitis-like syndrome in about 20% of FMF patients (predominantly M694V/M694V), etc.

We have shown that particular mutations have significant correlation with renal amyloidosis (RA). In frames of International Meta-FMF project we compared our data with the FMF morbidity among the other populations. We confirmed that M694V mutation is a high risk factor of RA in patients in Armenia, Israel, Lebanon, but not associated with RA in Turkey. M694V homozygous genotype of MEFV in FMF patients with RA is significantly higher than in patients without RA. The risk of male patients to develop RA is four times higher than that of female patients. SAA (Serum Amyloid A) a/a homozygous genotype is also associated with a seven-fold increased risk of developing RA, compared to other SAA1 genotypes. The presence of only one SAA1 a/a allele does not suggest an increased susceptibility to RA. In our cohort of FMF patients the adequate colchicine-therapy may delay RA progression. In a few cases, the effect of colchicine remains controversial. M694V homozygotes present a more severe phenotype and show a limited response to colchicine at the nephrotic stage of RA. In contrast, FMF patients with other genotypes still have a good chance to escape the nephrotic syndrome and to maintain renal function.

## Conclusion

As a result of our 16-year experience, the CMG holds the largest DNA Biobank of FMF (more than 18000 samples). The number of patients visiting CMG is dramatically increasing due to complex clinical and genetic examinations, assessment of efficiency of colchicine treatment, prognosis of development of complications, including renal amyloidosis, counseling of families, professional and public awareness. Genetic counseling of FMF patients and their families is performed for the disease risk estimation for future generations.

## Disclosure of interest

None declared

